# Magnetic resonance imaging in the tibial epiphyseal growth plate development of Wistar rat

**DOI:** 10.1186/1749-799X-9-39

**Published:** 2014-05-20

**Authors:** Denglu Yan, Yancheng Song, Bin Shen, Pengde Kang, Fuxing Pei

**Affiliations:** 1 Nanshan Hospital of Guangdong Medical College, Shenzhen 518052, People's Republic of China; 2 West China Hospital, Sichuan University, Chengdu 610065, People's Republic of China; 3 Third Hospital of Nanfang Medical University, Guangzhou 510630, People's Republic of China

**Keywords:** Kashin–Beck disease, T-2 toxin, KBD-affected feed, Rat

## Abstract

**Objective:**

This research aims to investigate magnetic resonance imaging (MRI) in the tibial epiphyseal growth plate development of Wistar rat.

**Methods:**

Fifty weanling Wistar rats were divided by a computerized blocking procedure into five groups. The rats received standard commercial feed with or without T-2 toxin additive, low-protein feed with or without T-2 toxin additive, and Kashin–Beck disease (KBD)-affected feed.

**Results:**

Compared with the control group rat, MRI showed localized epiphyseal plate swelling, rough appearance, and uneven signal on the tibia of rats fed with KBD-affected feed. Histology confirmed the epiphyseal plate degeneration seen by MRI, and the degenerative changes were characterized by abnormal distribution of chondrocytes with loss and clustering, cartilage fragmentation, and erosion in group E.

**Conclusions:**

The MR image of the rat epiphyseal plate is altered in the KBD model rats, and epiphyseal plate MRI appearance has been reproduced by using T-2 toxin and KBD-affected feed of epidemic district.

## Introduction

Magnetic resonance imaging (MRI) is extensively used for the evaluation and examination of various kinds of tissues in medicine and dentistry because it produces high-quality images of the soft tissues without ionizing radiation [1]. In particular, the alternations of normal cartilaginous epiphyses, physes, and metaphyseal marrow during growth have been evaluated extensively with MRI because precise imaging of these tissues with plain film radiographs and computed tomography scans is difficult [2]. In the metaphyseal region of individuals affected with Kashin–Beck disease (KBD), MRI may be the most reliable and useful imaging modality for evaluation of the epiphyseal plate, which is otherwise difficult to evaluate because of its anatomical complexity and small size [3].

Kashin–Beck disease, endemic mainly in certain areas of China, usually afflicts children between the ages of 5 and 13 years, and initial pathologic changes are multiple degenerative and necrotic lesions within the cartilage of the metaphysis [4-11]. The A'ba Autonomous Region of Sichuan province has high altitude and extremely severe weather conditions which affect the availability of natural resources and the use of land [12-14]. Due to high moisture content, the grains are particularly susceptible to mold and severe fungal infection [15]. Fusarium species of fungi produce a variety of mycotoxins, including fumonisins, fusarins, trichothecenes, zearalenone, and T-2 toxin. T-2 toxin belongs to a large group of trichothecene mycotoxins synthesized by various Fusarium molds which can infect raw agricultural materials, and is considered to be a major causative agent of KBD in humans [12,16-19].

Although it is clear that KBD usually involves the metaphysis in a growing bone and etiology shows that low T-2 toxin average intake is a major causative agent of KBD, there are few studies about this in the literature, and more studies in animal models are needed to demonstrate the pathogenesis of low T-2 toxin average intake in KBD [19-22]. The primary lesion of KBD involves the epiphyseal plate and the articular cartilage [23,24]. Although the ability of T-2 toxin to produce necrosis of the epiphyseal plate cartilage tissue, including pronounced DNA and decreased numbers of chondrocytes, is well documented [16,17], little is presently known about the epiphyseal plate developmental biology of this mycotoxin in Wistar rat with low T-2 toxin average intake [25]. To date, no paper investigating the epiphyseal plate of rats which have been fed with KBD-affected feed and those with low T-2 toxin average intake has been published. In this study, *in vivo* MRI was undertaken on the hind knee of Wistar rats which had been fed with KBD-affected feed and T-2 toxin to investigate epiphyseal plate developmental biology and pathogenesis.

## Materials and methods

### Animals and diets

Fifty weanling Wistar rats (half of them male and the other half female, 4 weeks of age, and with an average weight of about 65 g) were obtained from the Center of Laboratory Animals of Huaxi Hospital, Sichuan University, Chengdu, Sichuan, People's Republic of China. The rats' rooms were maintained at a temperature of 22°C with a relative humidity of 40%–70% and a 12-h light/dark cycle. All rats were grouped into five animals per box. The use of animals in this study was in accordance with the NIH publication 85-23 ‘Guide for the Care and Use of Laboratory Animals’ (NRC, 1996). Prior to initiation of dosing, all rats were quarantined for 1 week and evaluated for weight gain and any gross sign of disease or injury. After quarantine, the rats were divided by a computerized blocking procedure into five groups. Group A had nine rats which served as the control and received standard commercial feed without feed additive. Group B had 11 rats and received standard commercial feed with T-2 toxin by intragastric administration. Group C had 12 rats and received low-protein feed with T-2 toxin by intragastric administration. Group D had nine rats and received low-protein feed, and group E had nine rats and received KBD-affected feed (the food of KBD patients). All rats were fed with tap water throughout the experimental period.

T-2 toxin was purchased from Trilogy (Washington, MO, USA) in 99% pure desiccated form, and 40 mg of T-2 toxin was first dissolved in 2 ml alcohol and then dissolved in 100 ml normal saline. The T-2 toxin was administered intragastrically 5 days a week at 0.04 mg/kg/day. KBD-affected feed consisted of wheat and corn samples which were collected in October 2008 from KBD-affected families located in Rangtang county of the A'ba prefecture, Sichuan, People's Republic of China. Children with KBD were diagnosed in the early stage of the disease based on the national diagnosing criteria for KBD in China (National diagnosing criteria for Kashin–Beck disease in China (1995)) by X-ray films of the right hand. The commercial rat diet and KBD-affected diet were taken from the center of disease prevention of Sichuan (methods: GB/T5009.5, 14, 42, 90, 92, 93-2003). The KBD-affected diet was made based on the KBD-affected family's dietary pattern; protein content was 11.28% and selenium was 0.11 mg/kg. Meanwhile, protein content was 18.46% and selenium was 0.14 mg/kg in the commercial rat feed. At 4, 8, and 12 weeks, rat knee samples composed of the tibial epiphyseal plate and adjoining metaphyseal bone were harvested and immersed in 4% aqueous solution of buffered formaldehyde.

### MR imaging

At 4, 8, and 12 weeks, MR imaging was performed *in vivo* on the rats using a 3.0 T MR scanner (Philips Achieva, Philips, Amsterdam, Netherlands) in the sagittal and coronal planes with turbo spin echo (TSE) T1 weighting and T2 weighting, and the MR imaging features are shown in Table 1. The frequency encoding direction was perpendicular to the long axis of the bone to avoid chemical shift in the physis. At the end of this study, the images were blindly reviewed by two experienced radiologists. Epiphyseal plate appearances were evaluated qualitatively: the intensity variations were scored high, medium, or low based on comparison with muscle signal, which was assumed constant. In addition, epiphyseal plate swelling was assessed by direct comparison with the rats on normal diet.

**Table 1:** Philips Achieva 3.0 T MR system used to scan the rat samples

**Technique**	**T2WI TSE**	**T1WI TSE**	**T2WI TSE**
TR	4,000	500	4,000
TE	66	23	66
NSA	6	6	6
FOV	60 × 50	60 × 55	60 × 55
Matrix	172 × 150	180 × 259	172 × 162
Slice thickness (mm)	1	1	1
Gap (mm)	0.2	0.2	0.2
Fold-over direction	AP	RL	RL
Flip angle (deg)	90	90	90
Slice orientation	Sagittal	Coronal	Coronal
SPAIR	Fat suppression	No	Fat suppression
Total scan time	10:24	8:48	11:12

*TSE* turbo spin echo, *TR* repetition time, *TE* echo time, *NSA* number of signal averages, *SPAIR* spectral attenuated inversion recovery.

### Histological examinations

MRI findings were then compared with histological findings. The femora and tibiae were fixed in 4% paraformaldehyde, decalcified, and embedded in paraffin, and then 4-μm-thick longitudinal sections from the mid-portion of each bone were stained with hematoxylin and eosin (H&E) and counterstained with toluidine blue. Pathological changes were observed under an optical microscope (Olympus BX 41, Olympus, Tokyo, Japan) and assessments of epiphyseal plate necrosis [26,27].

### Statistical analyses

Statistical analyses were performed using SPSS Version 16.0 software. The data were expressed as mean ± SD and were analyzed using one-way analysis of variance (ANOVA) followed by LSD post hoc tests. Results were considered statistically significant if the *P* value was less than 0.05 for continuous variables.

## Results

There were no rat deaths in groups A, D, and E, while higher mortalities were associated with T-2 toxin: three in group B (2/11) and three in group C (3/12). The weight gain of the rats is shown in Table 2. The highest weight gain was in the control group, and the lowest was in the KBD feed group (*P* < 0.05). The T-2 toxin groups showed no difference at all three time points (*P* > 0.05), although the rats in the low-protein with T-2 toxin group had less weight gain than the rats on standard commercial feed with T-2 toxin. There was a significant difference for the T-2 toxin groups compared with the control group (*P* < 0.05), and the T-2 toxin groups with the KBD feed group (*P* < 0.05). The rats in the low-protein group had less weight gain than the rats in the control group at three time points (*P* > 0.05), and had more weight gain than the rats in the KBD feed group (*P* < 0.05).

**Table 2:** The weight gain data of rats (g) presented as mean ± SD

**Time point**	**Group A**	**Group B**	**Group C**	**Group D**	**Group E**
4 weeks	121.75 ± 18.23	75.33 ± 5.69	43.60 ± 7.20	85.50 ± 2.12	29.67 ± 2.55
8 weeks	234.25 ± 36.72	116.33 ± 8.08	107.50 ± 9.19	142.50 ± 6.36	55.25 ± 3.86
12 weeks	297.50 ± 62.24	209.50 ± 33.94	141.00 ± 40.31	222.00 ± 69.29	77.00 ± 24.07

The highest weight gain was in the control group, and the lowest was in the KBD feed group (*P* < 0.05).

**Figure 1:**
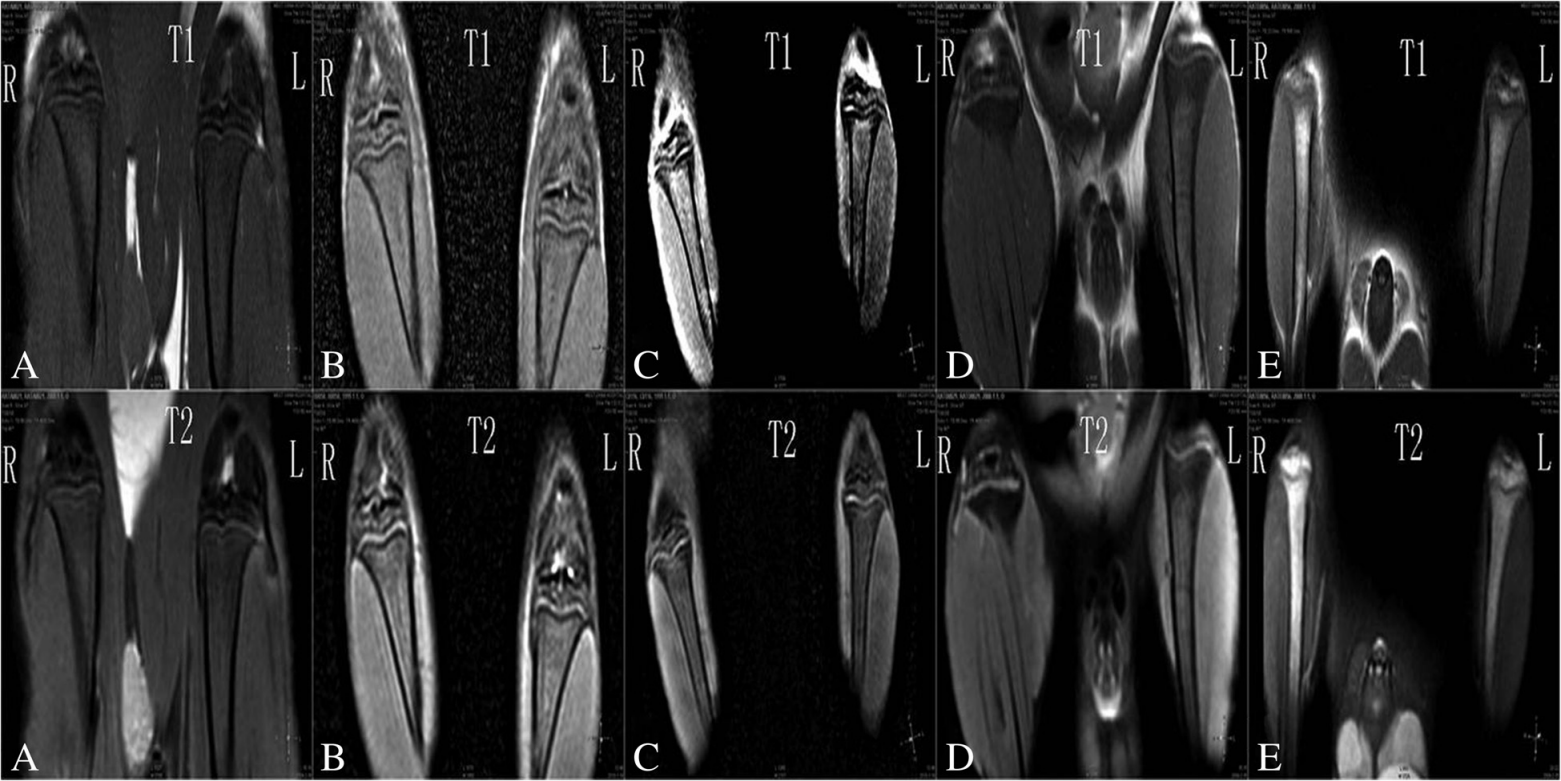
**MR images of the epiphyseal plate at 8 weeks. (A)** Epiphyseal plate *shows clear, wide, continuous and smooth; the intensity shows medium and uniformity*. **(B)** Epiphyseal plate* shows clear, narrow, continuous and smooth; the intensity shows medium and uniformity*. **(C)** Epiphyseal plate *shows narrow, disccontinuous and rough; the intensity shows higher or low and uneven*. **(D)** Epiphyseal plate *shows clear, wide, continuous and smooth; the intensity shows medium and uniformity*. **(E)** Epiphyseal plate *shows narrow, discontinuous and rough; the intensity shows higher or low and uneven*.

**Figure 2:**
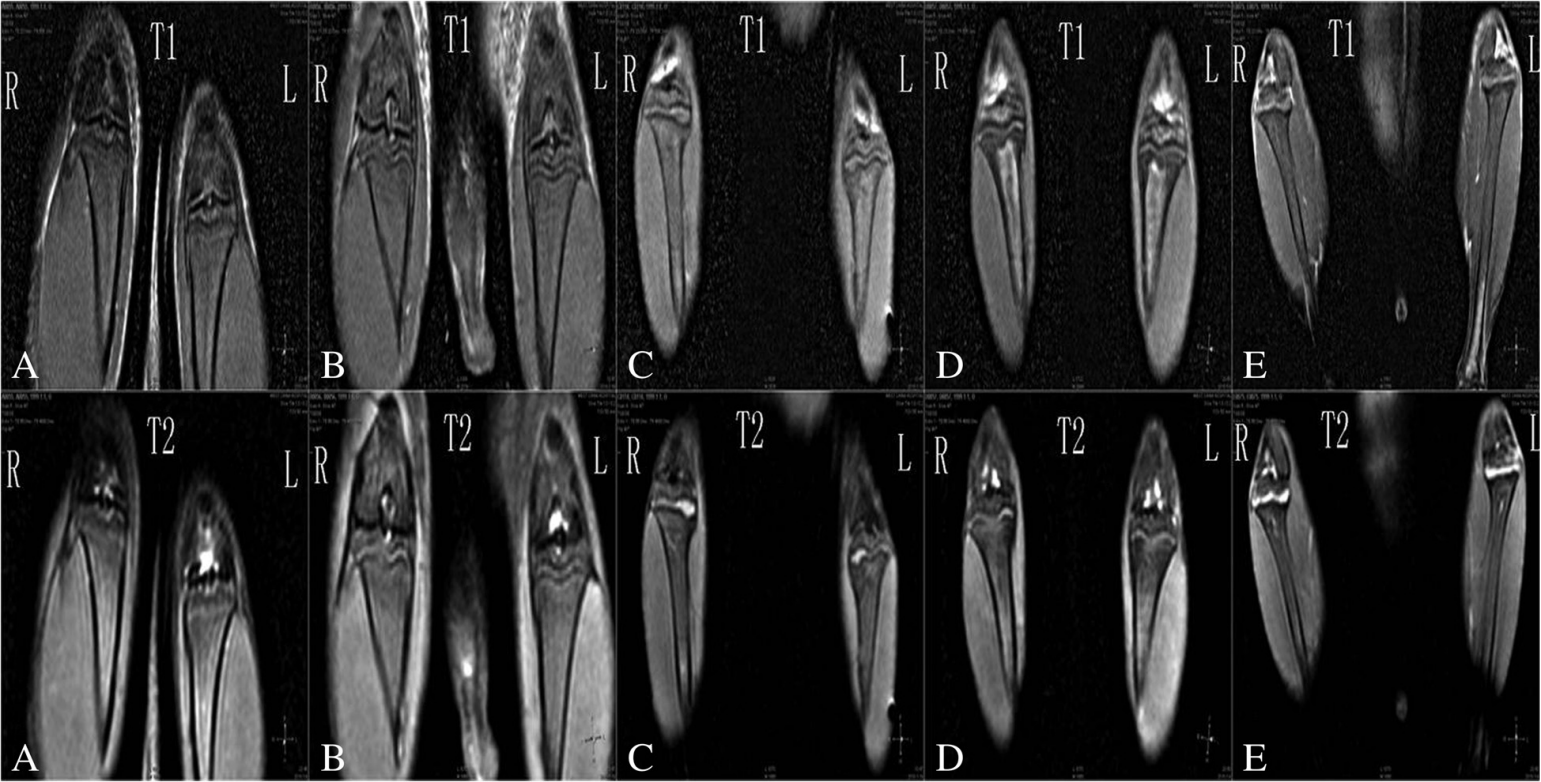
**MR images of the epiphyseal plate at 12 weeks. (A)** Epiphyseal plate *shows narrow, discontinuous and rough; the intensity shows low and uneven*. **(B)** Epiphyseal plate *shows narrow, discontinuous and rough; the intensity shows low and uneven*. **(C)** Epiphyseal plate *shows narrow, discontinuous and rough; the intensity shows higher or low and uneven*. **(D)** Epiphyseal plate *shows narrow, discontinuous and rough; the intensity shows low and uniformity*. **(E)** Epiphyseal plate *shows narrow, discontinuous and rough; the intensity shows higher or low and uneven*.

In the MR images of the rats in the control group at 4, 8, and 12 weeks, the epiphyseal plate appeared histologically normal and of smooth appearance and uniform signal (Figures 1 and 2 and Table 3). At 12 weeks, the MR images showed darkening on the epiphyseal plate and marked lower signal than at 8 and 4 weeks. Compared with rats in the control group, MRI showed localized epiphyseal plate swelling on the tibia of the KBD feed rats at 8 and 12 weeks. At 12 weeks, MR images showed rough appearance and uneven signal. The appearance of the epiphyseal plate of rats in groups B and D was similar to that of the control group. The epiphyseal plate of rats in group C was similar to that of the KBD feed group rats, which had lower signal than the KBD feed groups and higher than the other groups at 12 weeks.

**Table 3:** Epiphyseal plate appearances and intensity

**Group**		**Epiphyseal plate**	
		**Appearances**	**Intensity**
A	4 weeks	Clear, wide, continuous, smooth	High, uniform
	8 weeks	Clear, wide, continuous, smooth	Medium, uniform
	12 weeks	Narrow, discontinuous, rough	Low, uneven
B	4 weeks	Clear, wide, continuous, smooth	High, uniform
	8 weeks	Clear, narrow, continuous, smooth	Medium, uniform
	12 weeks	Narrow, discontinuous, rough	Low, uniform
C	4 weeks	Clear, wide, continuous, smooth	High, uniform
	8 weeks	Narrow, discontinuous, rough	High or low, uneven
	12 weeks	Narrow, discontinuous, rough	High or low, uneven
D	4 weeks	Clear, wide, continuous, smooth	High, uniform
	8 weeks	Clear, wide, continuous, smooth	Medium, uniform
	12 weeks	Narrow, discontinuous, rough	Low, uniform
E	4 weeks	Clear, wide, continuous, smooth	High, uniform
	8 weeks	Narrow, discontinuous, rough	High or low, uneven
	12 weeks	Narrow, discontinuous, rough	High or low, uneven

**Figure 3:**
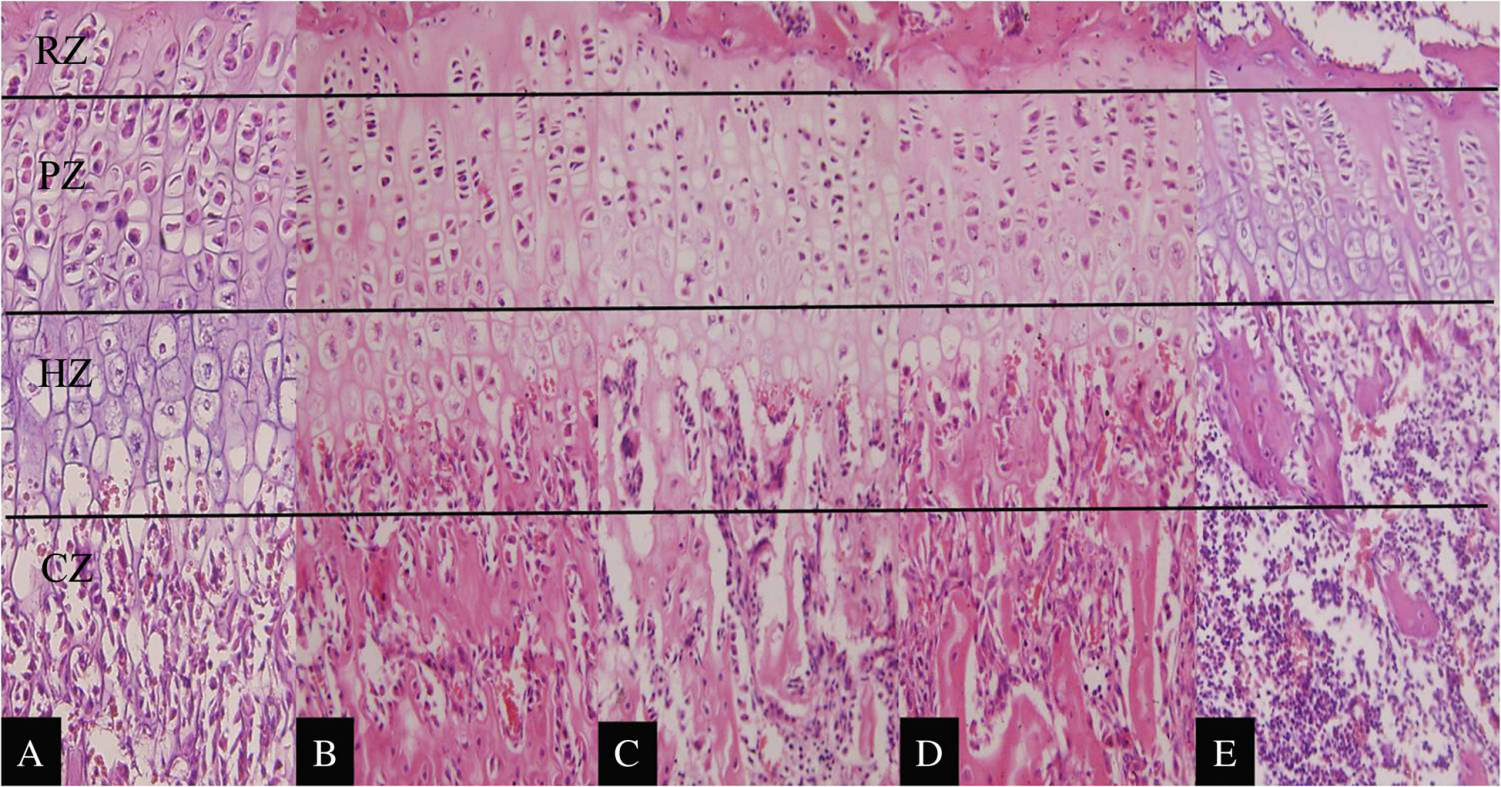
**H&E staining of rat epiphyseal plate cartilage at 12 weeks. (A)** No epiphyseal plate chondrocyte necrosis. **(B)** Chondrocyte necrosis in PZ and HZ. **(C)** Chondrocyte necrosis in PZ and HZ. **(D)** No epiphyseal plate chondrocyte necrosis. **(E)** Chondrocyte necrosis in PZ and HZ. RZ= Resting zone, PZ= Proliferation zone, HZ= Hypertrophy zone, CZ= Calcification zone.

H&E staining of rat epiphyseal plate cartilage is shown in Figure 3. There was no epiphyseal plate chondrocyte necrosis in group A at 4, 8, and 12 weeks. In the normal rat epiphyseal plate cartilage, extensive matrix and cellular lacunae can be clearly seen. However, in the epiphyseal plate cartilage of rats in groups B, C, and E, chondrocyte necrosis is seen in the lower hypertrophic layer (proliferation cell zone and labrocyte cell zone) at the three time points. Compared with the control group, histology confirmed the presence of these changes seen by MRI including epiphyseal plate thickening and degeneration in KBD feed rats. A representation of this change is shown in Figure 3, where erosion of the epiphyseal plate exists side by side with epiphyseal plate thickening in group E. There were areas of cartilage thickening, thinning, and loss with presence of residual matrix similar at 8 weeks, but more severe to degenerative changes seen at 12 weeks. At 12 weeks, histology confirmed the epiphyseal plate degeneration seen by MRI, and the degenerative changes were characterized by abnormal distribution of chondrocytes with loss and clustering, cartilage fragmentation, and erosion in group E.

## Discussion

The study on the etiology of KBD showed that the contamination of food by mycotoxins has been suggested as causal agents, which may act together with other environmental factors to increase hazard stress on the cartilage [8,15,28]. In this study, as would be expected, high mortalities were associated with the T-2 toxin group. The rat diet of KBD-affected feed was taken from the A'ba Autonomous Region of Sichuan province, and the grains are particularly susceptible to mold and severe fungal infection due to their high moisture content. The pathology of Kashin–Beck disease may be explained by the effect of mycotoxins, and different species of fungi produce a wide range of mycotoxins [12,25]. Although T-2 toxin is usually ingested orally to test for specific bone malformations associated with KBD, a cartilage growth defect with reduced thickness of the epiphyseal plate caused by the attenuation of the proliferative zone, it is particularly interesting whether KBD-affected feed impaired the Wistar rats' epiphyseal plate.

MRI is a powerful technique to investigate this as it is sensitive and suited to detect compositional change and integrity of the bone, cartilage, and epiphyseal plate. MRI is extensively used for the evaluation and examination of various kinds of tissues in medicine and dentistry because it produces high-quality images of the soft tissues without ionizing radiation [1,2]. In the metaphyseal region of KBD patients, MRI may be the most reliable and useful imaging modality for evaluating the epiphyses plate, which is otherwise difficult to evaluate because of its anatomical complexity and small size [3,29]. KBD affects only the growing bone, which is formed through endochondral ossification [30]. In this study, the MR images of rats at 12 weeks showed the presence of darkening and marked low signal than at 8 and 4 weeks on the epiphyseal plate of rats in the control group, but the epiphyseal plate appeared histologically normal and of smooth appearance and uniform signal. Compared with the control group rat, MRI showed localized epiphyseal plate swelling on the tibia of KBD feed rats at 8 and 12 weeks. At 12 weeks, MR images showed a rough, hard, spherical appearance and uneven signal.

The primary lesions of KBD involve the epiphyseal plate and articular cartilage [23,24]. Although the ability of T-2 toxin to produce necrosis of epiphyseal plate cartilage tissue, including pronounced DNA and decreased numbers of chondrocytes is well documented [16,17], little is presently known about the epiphyseal plate developmental biology of this mycotoxin in Wistar rats with low T-2 toxin average intake [25]. To date, there has been no paper published which investigates the epiphyseal plate of rats after having been fed with KBD-affected feed and low T-2 toxin average intake. We compared the MR appearance and histology of the tibial epiphyseal plate. Histological examinations demonstrated that the expanding low-intensity area on T1- and T2-weighted images corresponded to the expanding bone marrow cavity and primary spongiosa. Histology confirmed the presence of these changes including epiphyseal plate thickening and degeneration.

## Conclusions

The rat epiphyseal plate is altered on MRI in the KBD model rats, and the epiphyseal plate MRI appearance has been reproduced by use of T-2 toxin and KBD-affected feed of the epidemic-affected district in the A'ba autonomous region.

## Competing interests

The authors declare that they have no competing interests.

## Authors’ contributions

DY participated in the design of the study and drafted the manuscript. YS, BS, and PK participated in the design of the study and helped draft the manuscript. FP participated in the design and coordination of the study and helped draft the manuscript. All authors read and approved the final manuscript.
